# The Crusade against Consumption

**Published:** 1898-11-12

**Authors:** 


### THE CRUSADE AGAINST CONSUMPTION.

Amid all that is being urged, in public addresses and otherwise, as to the importance of taking special measures to suppress tuberculosis, it will not do to forget how steadily the mortality from consumption has been diminishing during recent years. If we take the figures given in the Report of the Royal Commission on Tuberculosis, we find that, comparing the average of the ten years 1861-70 with that of the five years 1890-95, the death-rate in England and Wales from all forms of tuberculous disease in the former period was 3,240 per million living, while in the latter period it was only 2,122 per million living. This diminution of mortality is curiously regular and progressive, showing that long before any special measures for the suppression of tuberculous disease had come even into men's minds, not to mention their having come into operation, the disease had been gradually giving way before the steady advance which during the same period had been made in general sanitation. This is really a very important point to bear in mind, however thoroughly we endorse all that is being urged in the way of a "special crusade" against the disease. Inspection of meat, and especially inspection of milk, is good; the provision of asylums for the hopelessly consumptive and of institutions for the treatment of those who may perchance be curable is good also, if only for mere pity's sake; but let us not forget that, while all the modes of infection at the suppression of which these measures aim have been in full operation, the disease has been steadily diminishing under the influence of a gradual although very slow improvement in the standard of living and in the sanitary surroundings of the people. Fresh air, free ventilation, cleanliness, and a diminution of that overcrowding which makes cleanliness, decency, and ventilation well-nigh impossible for a large proportion of our population, are the true preventives of tuberculous diseases. Whether in regard to humans, or to cattle, or to wild beasts in captivity, consumption is the curse which specially attaches to those who dwell in houses. The great social problem for the coming century to solve is how to keep a roof over our heads, and yet avoid this disease. Civilisation without tuberculosis would, indeed, be a social revolution.

